# Draft genome of *Raoultella planticola*, a high lead resistance bacterium from industrial wastewater

**DOI:** 10.1186/s13568-023-01519-w

**Published:** 2023-01-30

**Authors:** Nagwa I. Elarabi, Asmaa A. Halema, Abdelhadi A. Abdelhadi, Ahmed R. Henawy, Omar Samir, Heba A. R. Abdelhaleem

**Affiliations:** 1 Genetics Department; Faculty of Agriculture, Cairo University, Giza, 12613 Egypt; 2 National Biotechnology Network of Expertise (NBNE), Academy of Scientific Research and Technology (ASRT), Cairo, Egypt; 3 Department of Microbiology; Faculty of Agriculture, Cairo University, Giza, 12613 Egypt; 4 Genomic Research Program, Children’s Cancer Hospital, Cairo, Egypt; 5 Biotechnology College, Misr University for Science and Technology (MUST), 6(th) October City, Egypt

**Keywords:** Bioremediation, Lead resistant bacteria, Whole-genome sequencing, Illumina Miseq, *Raoultella planticola*

## Abstract

Isolation of heavy metals-resistant bacteria from their original habitat is a crucial step in bioremediation. Six lead (Pb) resistant bacterial strains were isolated and identified utilizing *16S rRNA* to be *Enterobacter ludwigii* FACU 4, *Shigella flexneri* FACU, *Microbacterium paraoxydans* FACU, *Klebsiella pneumoniae* subsp. *pneumonia* FACU, *Raoultella planticola* FACU 3 and *Staphylococcus xylosus* FACU. It was determined that all these strains had their Minimum inhibitory concentration (MIC) to be 2500 ppm except *R. planticola* FACU 3 has a higher maximum tolerance concentration (MTC) up to 2700 ppm. We evaluated the survival of all six strains on lead stress, the efficiency of biosorption and lead uptake. It was found that *R. planticola* FACU 3 is the highest MTC and *S. xylosus* FACU was the lowest MTC in this evaluation. Therefore, transmission electron microscopy (TEM) confirmed the difference between the morphological responses of these two strains to lead stress. These findings led to explore more about the genome of *R. planticola* FACU 3 using illumine Miseq technology. Draft genome sequence analysis revealed the genome size of 5,648,460 bp and G + C content 55.8% and identified 5526 CDS, 75 *tRNA* and 4 *rRNA*. Sequencing technology facilitated the identification of about 47 genes related to resistance to many heavy metals including lead, arsenic, zinc, mercury, nickel, silver and chromium of *R. planticola* FACU 3 strain. Moreover, genome sequencing identified plant growth-promoting genes (PGPGs) including indole acetic acid (IAA) production, phosphate solubilization, phenazine production, trehalose metabolism and 4-hydroxybenzoate production genes and a lot of antibiotic-resistant genes.

**Supplementary Information:**

The online version contains supplementary material available at 10.1186/s13568-023-01519-w.

## Introduction

Due to industry’s rapid development, pollution of water and soil environments by heavy metals has increased in many countries, leading to serious environmental problems including environmental pollution and ecological degradation (Briffa et al. 2020). Heavy metals can become strongly toxic by mixing with different environmental elements, such as water, soil, air, and living organisms that can be exposed to them through the food chain which are considered the primary ecological challenge of human life (Mitra et al. 2022). These heavy metals enter natural ecosystems through human resources and natural processes particularly industrial and agricultural such as lead melting, lead painting, pottery, grids, textiles, battery recycling, arms production, book printing, pigments, coal and petroleum burning, microelectronics, nuclear power stations, plastics, and wood preservation (Kohzadi et al. 2018; Minari et al. 2020; Krivokapić 2021). Some of the heavy metals present in low concentrations are essential factors in human, animals and plant physiology but if present in higher concentrations can be toxic (Kohzadi et al. 2018). The appearance of heavy metals more than the standard in the environment Pb to negative effects on human, animals, plants and the environment (Kanagaraj et al. 2014; Sharma et al. 2017). Heavy metals can cause many ecosystem problems due to the non-biodegradable ability of heavy metals which can cause many diseases (Guo et al. 2019; Boushehrian et al. 2020).

Several physical–chemical techniques such as ion exchange, precipitation, coagulation, adsorption and electrochemical methods have been utilized to separate heavy metal ions from different sources (Wu et al. 2019). The environment contamination, toxic ions production, high cost, and ineffectiveness at low concentrations are among the disadvantages of utilizing these methods (Tamjidi et al. 2019). Therefore, it is important to decontaminate potentially toxic ions in a contaminated environment, and utilizing microorganisms is one of the useful methods. A process to use bacteria to facilitate toxic ions removal or to convert them to less harmful compounds from an environment site is known as bioremediation (Elarabi et al. 2020; Krastanov et al. 2013). Bioremediation methods are valuable in comparison with physical–chemical techniques for heavy metal removal methods due to the high effectiveness of these methods at low metal concentrations. In addition, bioremediation methods are eco-friendly, less disruptive, economical, field-scale application, low time of remediation and high public acceptability (Bogdanova et al. 1992; Khalid et al. 2017). Bacteria are currently utilized in several pollutants bioremediation such as polyaromatic hydrocarbon (Abdelhaleem et al. 2019), pesticides (Elarabi et al. 2020) and heavy metals (Mohamed et al. 2020; González and Ghneim-Herrera 2021). Biological method of remediation of polluted sites uses living organisms, which could be plant (phytoremediation) or microorganisms (bioremediation) such as bacteria, algae and fungi, or their products to abate or clean up pollutants (Alori et al. 2022). One of the best favorable bioremediation techniques is bacterial remediation, which utilizes bacteria to enhance biosorption, precipitation, intracellular accumulation and oxidation. Some bacteria contain different genes involved in heavy metals resistance that permit them to adapt to the heavy metal high concentrations (Khan et al. 2009; Bhagat et al. 2016), thus they may be utilized as absorbing agents to remove pollutants (Abdelatey et al. 2011). Bacteria have many different mechanisms of metal resistance such as volatilization via ethylation or methylation, precipitation of metals as phosphates, energy-dependent metal efflux systems, extra cellular polymeric substances (EPS) and membranes physical exclusion of ions (Silver 1996).

Bacteria help to detoxify heavy metals in the environment. Detoxification can occur through the valence transformation mechanism. This is particularly applicable in the case of metals whose different valence states vary in toxicity. In mercury-resistant bacteria, organomercurial lyase converts methyl mercury to Hg(II), which is one 100-fold less toxic than methyl mercury (Wu et al. 2010). The reduction of Cr(VI) to Cr(III) that having less mobility and toxicity. Other detoxification mechanisms of heavy metals are accomplished through metal binding, vacuole compartmentalization, and volatilization. Metal binding involves chelators, such as metallothein, phytochelatin, and metal binding peptides. These chelators bind to heavy metals and facilitate bacterial absorption and the transportation of metal ions. Volatilization mechanisms involve turning metal ions into a volatile state. This is only possible with Se and Hg, which have volatile states (Wu et al. 2010). The reduction of Se(V) to Se(0) has been employed to remediate contaminated waters and soils. The metabolic processes of these organisms help to transform pollutants in the environment (Siddiquee et al. 2015).

Arsenic (As), cadmium (Cd), mercury (Hg) and lead (Pb) possess no useful influence on human, animal and plant and they are considered even toxic (Adriano 2001). Pb is considered a main pollutant that is found in air, soil and water. This metal is also dangerous waste and extremely toxic to any living organism (Low et al. 2000). Pb also causes damage to cell proteins especially the metabolism enzymes, cell membranes and carcinogenesis (Olaniran et al. 2013). In addition, Pb toxicity causes different symptoms in the hepatic, nervous system, (Flora et al. 2012) and interacts with the genetic material, by binding to fundamental transcription factors (Vallee and Ulmer 1972).

Bacteria respond to excess Pb by metal-inducible resistance mechanisms. Pb bacterial resistance is fundamentally based on metal ions active efflux to prohibit its harmful effects in the cell (Rensing et al. 1998). Pb resistance is slightly less studied, but Pb detoxification and P-type ATPases are known. In addition, Pb low-level resistance is performed by binding Pb ions in the inactive form including the nonspecific binding to the cell wall and metal-inducible binding factors. Three main families of efflux transporters are implicated in Pb resistance. Capsule biogenesis assembly (CBA) transporters work as chemiosmotic antiporters in Gram negative bacteria (Franke et al. 2003), P-type ATPases pump Pb ions outside the cell from the cytoplasm (Rensing et al. 1998) and cation diffusion facilitator (CDF) transporters perform as chemiosmotic ion-proton exchangers (Grass et al. 2001). CDF transporters and P-type ATPases are generally found in several species of bacteria, while CBA transporter is extraordinary and showed high-level resistance to Pb ions (Nies 2003). In addition, some bacteria possess binding factors which detoxify Pb by sequestration. These binding factors contain exopolysaccharides (cell wall components) and metallo-chaperones (intracellular binding proteins) (Nies 2003).

Many bacterial species utilize Pb extra and intracellular binding to avoid toxicity. *Staphylococcus aureus* (Levinson et al. 1996), *Citrobacter freundii* (Levinson and Mahler 1998), *Bacillus megaterium* (Roane 1999) and *Vibrio harveyi* (Mire et al. 2004) minimize the Pb concentration as a phosphate salt utilizing precipitation. *Pseudomonas marginalis* precipitate Pb as an extracellular polymeric to avoid its toxicity (Roane 1999). However, the Pb precipitation molecular mechanisms for these bacteria are not understood. Several bacteria possess an envelope or cell wall that is eligible for adsorbing high levels of dissolved metals, commonly by a charge-mediated attraction (Mohamed 2001). The binding of Pb takes place fundamentally through exopolysaccharides (EPSs) in these bacteria (Loaec et al. 1997).

Many Pb resistant bacteria have been isolated from Pb contaminated industrial wastewater and soil including Gram-positive bacteria such as *Bacillus cereus, Bacillus sp*., *Arthrobacter* sp. and *Corynebacterium* sp. (Trajanovska et al. 1997; Zanardini et al. 1997; Shin et al. 2012) and the Gram-negative bacteria such as *Pseudomonas fluorescens*, *Pseudomonas marginalis* (Hasnain et al. 1993), *Enterobacter* sp. and *Pseudomonas vesicularis* (Roane and Kellogg 1996; Sheng et al. 2008). There are some fungi like *Penicillium* sp. Psf-2, *Saccharomyces cerevisiae* and *Rhodotorula mucilaginosa* which is efficient in Pb bioadsorption (Sun and Shao 2007; Chatterjee et al. 2011). *Raoultella* is classified as *Enterobacteriaceae* family and is Gram*-*negative aerobic *bacilli* (Luo et al. 2017) that was initially part of the Klebsiella genus, but later reclassified utilizing the *16S rDNA, rpoB, gyrA* and *gyrB* genes (Drancourt et al. 2001). *Raoultella planticola* is an oxidasenegative non-motile (Drancourt et al. 2001). This bacterium used to be considered an environmental organism residing in water and soil.

Therefore, understanding the mechanisms of metal resistance and impact of heavy metals on bacteria are essential and important in order to remove the heavy metals from polluted environments. With the rapid development of next-generation sequencing technology, whole-genome information has been obtained for many microorganisms and plants. Whole genome sequencing (WGS) is a low cost, fast and highly effective technology that can provide complete information about the bacterial genome sequence. The differences between species can be better identified utilizing WGS and by following gene annotations utilizing online databases, such as kyoto encyclopedia of genes and genomes (KEGG), gene ontology (GO), non-redundant (NR) and clusters of orthologous genes (COG). WGS has become a widespread detection technique and is vastly utilized to identify microbial communities within intestinal flora, fungi and soil (McDermott et al. 2016; Tyler et al. 2018). It is necessary for mining the core genome, analyzing functional genomics and identifying specific genes, which ultimately contributes to the exploration of the diversity and biological characteristics of unknown microbial groups (Ronholm et al. 2016). The aims of the current study were to isolate, identify, and describe some Pb-resistant bacteria from heavy metal polluted samples to obtain strains that could be appropriate for the immobilization and detoxification of heavy metals in contaminated environments. In addition, in the present study the genome of *Raoultella planticola* FACU 3 strain was sequenced and analyzed in detail, as well as heavy metal resistance genes and genomic potentials were characterized. *R. planticola FACU 3* draft genome was obtained to study the endophytic characteristics of this bacterium at the genetic level.

## Material and methods

### Samples collection and measurement of physicochemical parameters

Three samples were collected from different heavy metals contaminated locations. The first location was from wastewater and sediment sample from Al-Rahawy drain, Giza Governorate, Egypt (30°12′16.3″N 31°02′03.5″E) and the second location was from industrial wastewater sample from 4th industrial zone, Borg Elarab city, Alexandria Governorate, Egypt (30°50′56.0″N 29°36′42.0″E). Industrial wastewater sample and sediment sample were collected during the period from September to November 2019. The three samples were stored at 4 °C until analysis. The physicochemical parameters of the collected samples were measured. The sample’s pH was determined (1:2.5 v/v for wastewater samples or 1:2.5 w/v for sediment sample) by digital pH meter; electrical conductivity was estimated (1:2.5 v/v for wastewater samples or 1:2.5 w/v for sediment sample) by conductivity meter. The concentrations of arsenic (As^2+^), cadmium (Cd^2+^), chromium (Cr^2+^), copper (Cu^2+^), iron (Fe^2+^), manganese (Mg^2+^), nickel (Ni^2+^), lead (Pb^2+^) and zinc (Zn^2+^) in the three samples were measured with an atomic absorption spectrophotometer (Buck Model 210 VGP). All analyses were performed in triplicate.

### Isolation of lead resistant bacteria

Pb stock solution (20%) was made by adding 20 g from C_4_H_6_O_4_Pb in 100 ml double distilled water and sterilized by utilizing 0.22 μm sterile syringe filters. The other concentrations were performed by dilution from the above stock solution. Isolation and enumeration of the Pb resistance bacterial were performed utilizing serial dilution method (1 mL of wastewater samples were suspended in 9 ml of sterile distilled water (dH_2_O) and serially diluted to 10^–6^ with dH_2_O and for the sediment, 1 gm from the sample was disrobed in 100 ml sterilized dH_2_O (Ben-David and Davidson 2014). Then, 0.1 ml of diluted suspension was added to Luria Bertani (LB) media (Peptone 10.00 g/L, NaCl 10.00 g/L, yeast extract 5.00 g/L and agar 20.00 g/L: pH 7.00) supplemented with (50, 100, 250, 500, 1000 and 1200) mg/L C_4_H_6_O_4_Pb (Lin et al. 2016). The plates were incubated at 30 °C for 3–15 days.

### Pb minimum inhibitory concentration (MIC) and higher maximum tolerance (MTC) determination

MIC and MTC of Pb resistance bacterial isolates were measured utilizing the agar plate dilution method (Malik and Jaiswal 2000). Different concentrations (1200–2500 mg/L) of C_4_H_6_O_4_Pb were added to sterilize LB plates which were then inoculated with bacterial isolates. The plates were incubated for 15 days at 30 °C.

### Survival and suppression percentage under Pb condition

To determine the increasing/decreasing of the bacterial count under Pb high concentration, Survival and suppression percentage of the bacterial isolates were measured utilizing the Colony-Forming Unit (CFU) after 15 days. Bacterial isolates were incubated on LB media with and without 2500 mg/L of C_4_H_6_O_4_Pb (the MTC concentration) and incubated at 30 °C for 15 days under shaking (150 rpm/min.). After that serially diluted to 10^–6^ with sterilized dH_2_O were performed. Then, 0.1 ml of diluted suspension was placed on free LB solid media. CFUs of the bacterial isolates were carried out utilizing the spread plate methods (Sanders 2012).

The survival and suppression percentages were determined through the following equations:

$$ {\text{Suppression percentage}} = \, \left( {{\text{CFU}}_{{{\text{control}}}} - {\text{ CFU}}_{{{\text{treatment}}}} } \right)/{\text{ CFU}}_{{{\text{control}}}} *{1}00 $$

$$ {\text{Survival percentage }} = {1}00 - {\text{suppression percentage}} $$

### Determination of Pb biosorption capacity and Pb uptake

To determine the Pb removal rates by the bacterial isolates, LB media supplemented with 2500 mg/L of C_4_H_6_O_4_Pb were performed. The suspension of selected isolates, for which the OD_600_ value was adjusted to 1.0, was inoculated into 25 mL LB medium and incubated at 30 °C with shaking at 150 rpm/min. for 15 days. The treated bacterial cultures were centrifuged at 5000×*g* for 20 min. The harvested cells were washed twice with dH_2_O and dried at 80 °C for 48 h in an oven. Then, bacterial dry weight was estimated. The residual Pb ion concentration was measured in the supernatants utilizing inductively coupled plasma atomic emission spectroscopy (ICP-AES) (as mg/L). The amounts of Pb uptake (mg/L) and Pb biosorption percentage were calculated utilizing the equation of Shetty and Rajkumar (2009):

$$ {\text{Pb}}^{{}} {\text{uptake }}\left( {{\text{mg}}/{\text{L}}} \right) = {\text{V }}\left( {{\text{CI}} - {\text{CF}}} \right)/{\text{dry biomass weight }}\left( {\text{g}} \right) $$

$$ {\text{Efficiency of biosorption }}\left( \% \right) \, = \, \left( {\left( {{\text{CI}} - {\text{CF}}} \right)/{\text{CI}}} \right)*{1}00 $$

where: V: volume of reaction; CI: Initial Pb concentration; CF: Final Pb concentration (Residual concentration).

### Molecular identification

The most possibility selected isolates for Pb resistance that showed the highest MIC value were initially identified to genus level via Gram staining, colony morphology, motility and laboratory biochemical tests including tests of urease, catalase, oxidase, methyl red, indole production, Voges Proskauer and different types of sugars fermentation ability (Sneath et al. 1986). For molecular conformation utilizing universal *16S rRNA* gene, genomic DNA was isolated utilizing Simply™ Genomic DNA Isolation Kit (Gene Direx, Inc. cat. no. SN023-0100, Taiwan) utilizing the manufacturer's instructions. Two universal primers [27F (5′-AGAGTTTGATCCTGGCTCAG-3′) and 1492R (5′- GGTTACCTTGTTACGACTT-3′)] for *16S rRNA* gene were used (Abdelhadi et al. 2016). PCR reaction proceeded in 50 μL total volume, containing 50 ng/μL DNA (5 μl), 2X (25 μL) One PCR™ master mix (Gene Direx, cat. no. MB203-0100, Taiwan), 10 pMOL (2.5 μL) from each primers and 15 μL nuclease-free water. PCR conditions, denaturation step for 5 min at 94 ºC and 40 cycles including denaturation for 1 min at 94 ºC; annealing for 1 min at 58 ºC and extension for 2 min at 72 ºC, then final extension step for 5 min at 72 ºC. Two percentage agarose gel were utilized for amplified products visualization under ultraviolet (UV) light. PCR product purification was performed utilizing ExoSAP-IT™ PCR Product Cleanup Reagent (Applied Biosystems, USA, cat. no 78201). The purified DNA was sequenced at Sangon Biotech Co., Ltd, Macrogen, Korea. The *16S rRNA* gene sequences of the isolates and their closely related strains were aligned together by ClustalOmega version 1.2.4 (Madeira et al. 2019). The sequence of the bacterial isolates were submitted to the GenBank database and compared with published sequences in the same database utilizing the NCBI BLAST program (http://www.ncbi.nlm.nih.gov/BLAST/) then confirmed by utilizing EzBioCloud DB software (https://www.bioiplug.com/). The alignment was trimmed with trimAl version 1.4.rev22 (Capella-Gutiérrez et al. 2009). Highly homologous sequences were selected and aligned utilizing CLUSTAL OMEGA. Phylogenetic tree was constructed by MEGA 11 utilizing the Maximum Likelihood method under the Kimura 2-parameter model, Bootstrapping was performed on 1000 bootstrap replications to assess the data.

### Transmission Electron Microscopy (TEM)

TEM were carried out to study the location of metal accumulations, as well as possible structural changes occurring in metal treated cells in comparison to untreated cells. Pb treated and untreated bacteria samples were performed according to Díaz et al. (2020). Thin sections (90 nm) were cut utilizing an Ultracut ultramicrotome (Leica UC7 Microsystems, Vienna, Austria) (Burghardt and Droleskey 2006). Sections were observed on a Tecnai G 20 TEM (FEI, Limeil-Brevannes, France) SA × 9900 at 200 kV (40000×). CCD camera was utilized for mages in conjunction with image processing software, iTEM of Olympus Soft Imaging System, Germany.

### Genome sequencing, molecular and phylogenetic analyses

The genomics DNA is extracted utilizing the QIAamp^®^ DNA Mini kit (QIAGEN, cat. no. 51304, Germany) following the manufacturer’s instructions. The genome sequencing of *R. planticola* FACU 3 was carried out using Illumina MiSeq™ platform (Illumina, USA) with a minimum of 1 Gb sequencing depth per sample by Genomics Research Program children’s Cancer hospital-Egypt 57,357 utilizing NextGen High Throughput Sequencing. Thus, a standard Illumina shotgun library “Nextera XT DNA Library Prep” was constructed and sequenced utilizing the Illumina MiSeq technology by synthesis. A paired-end sequencing strategy was utilized with an average size of 2 × 300 bp in length generated and a total number of reads of 25,425,220 bp obtained. The fastp v0.12.4 tool was utilized for evaluate the quality control of the raw data (Chen et al. 2018). SPAdes v3.13.1 was used for the filtered reads assembled (Bankevich et al. 2012). The assessment of the assembled files was carried out with QUAST v5.2.0 (Gurevich et al. 2013). Previously filtered reads were mapped to the reference utilizing BWA v 0.7.17−r1188 (Li and Durbin 2009). Variant identification and filtration were done utilizing BCFtools v 1.9 and SAMtools v 1.7 (Li 2011; Li et al. 2009). Most similar sequences were identified utilizing BLAST v 2.12.0 + and multiple sequence alignment was carried out utilizing MAFFT v7.505 (Katoh et al. 2002), followed by maximum likelihood phylogenetic tree generation utilizing IQ-TREE v 1.6.12 with 1000 bootstrap replications (Nguyen et al. 2015). GTR1F1I1G4 model calculation was done. The phylogenetic tree visualization was performed utilizing iTol (Letunic and Bork 2021). The gene annotation was carried out utilizing RAST (Aziz et al. 2008), Prokka v 1.14.6 (Seemann 2014) and Bakta v1.5.1 (Schwengers et al. 2020). PATRIC service was utilized for comprehensive genome analysis reads (Wattam et al. 2017). Antibiotic resistance genes annotation was assessed utilizing PATRIC’s genome annotation service (Wattam et al. 2017). The Resistance Gene Identifier (RGI) v5.1.1 tool of the Comprehensive Antibiotic Resistance Database (CARD) was utilized for *R. planticola* FACU 3 resistome analysis, where partial genes were excluded, and the predictions were made with contigs > 20,000 bp (McArthur et al. 2013). Abricate v0.8.13 (https://github.com/tseemann/abricate), PlasmidFinder v2.1.6 (Carattoli et al. 2014), PLATON v1.6–1 (Schwengers et al. 2020) were utilized to identify plasmids in the assembled genome.

### Statistical analysis

One way Analysis Of Variance (ANOVA) and Least Significant Difference (LSD) tests were performed using GraphPad Prism 8 and R respectively.

## Results

### Physicochemical analysis of collected samples

The industrial zone of Borg Elarab city is considered from the largest industrial cities in Egypt. The main industries and companies in these industrial zones are mining raw materials, batteries, plumbing, electric cables, electronic instruments and ceramic glazes. In addition, the sewage station of Al-Rahawy drain is considered one of the biggest industrial sewage stations that many industrial factories drain on it. In order to estimate the quality of the collected samples, physicochemical parameters such as pH, electric conductivity (EC) and heavy metal contents were evaluated (Additional file 1: Table S1). The pH was observed somewhat acidic ranged from 6.4 to 6.8 while the EC was noticed as low conductivity from 0.95 to 1.6. The concentrations of the toxic metals (Cd, As, Cu, Cr, Mg, Fe, Pb, Ni and Zn) in the collected samples were measured. The results showed that the heavy metal concentrations were higher than the United States Environmental Protection Agency (US EPA) screening standards for all the tested metals (US EPA 2022). The results displayed that El Rahawy drain sediment sample was higher in Cr, Cu, Fe, Mn, Ni, Pb and Zn while the 4th industrial zone, Borg Elarab sample was higher only in As and Cd (Additional file 1: Table S1). The Pb concentration in El Rahawy drain sediment (6.25 mg/Kg) was higher than that found in both El Rahawy drain wastewater (0.001 mg/L) and Borg Elarab sample (0.7 mg/L).

### Isolation of Pb resistant bacteria

Thirty bacterial isolates (L1–L30) were obtained and purified from the three samples. Those isolates were capable to grow in LB media supplemented with 1200 mg/L C_4_H_6_O_4_Pb. The Pb concentrations were gradually increased to determine MIC and MTC for these isolates. From these bacterial isolates, four isolates (L3, L4, L7 and L17) were isolated from El Rahawy drain sample and two isolates (L8 and L16) from 4th industrial zone, Borg Elarab sample showed the highest level of MIC and MTC (2600 ppm and 2500 ppm, respectively) as shown in Additional file 1: Fig. S1. These results suggested that those six isolates were capable of Pb resistance at different concentrations. Especially, L16 isolate was the best isolate that displayed a high ability to Pb resistance. L16 isolate was the higher bacterial isolates in both MIC and MTC (2800 and 2700 ppm respectively).

### Molecular identification of lead resistant bacteria utilizing *16S rRNA* gene

**Fig. 1:**
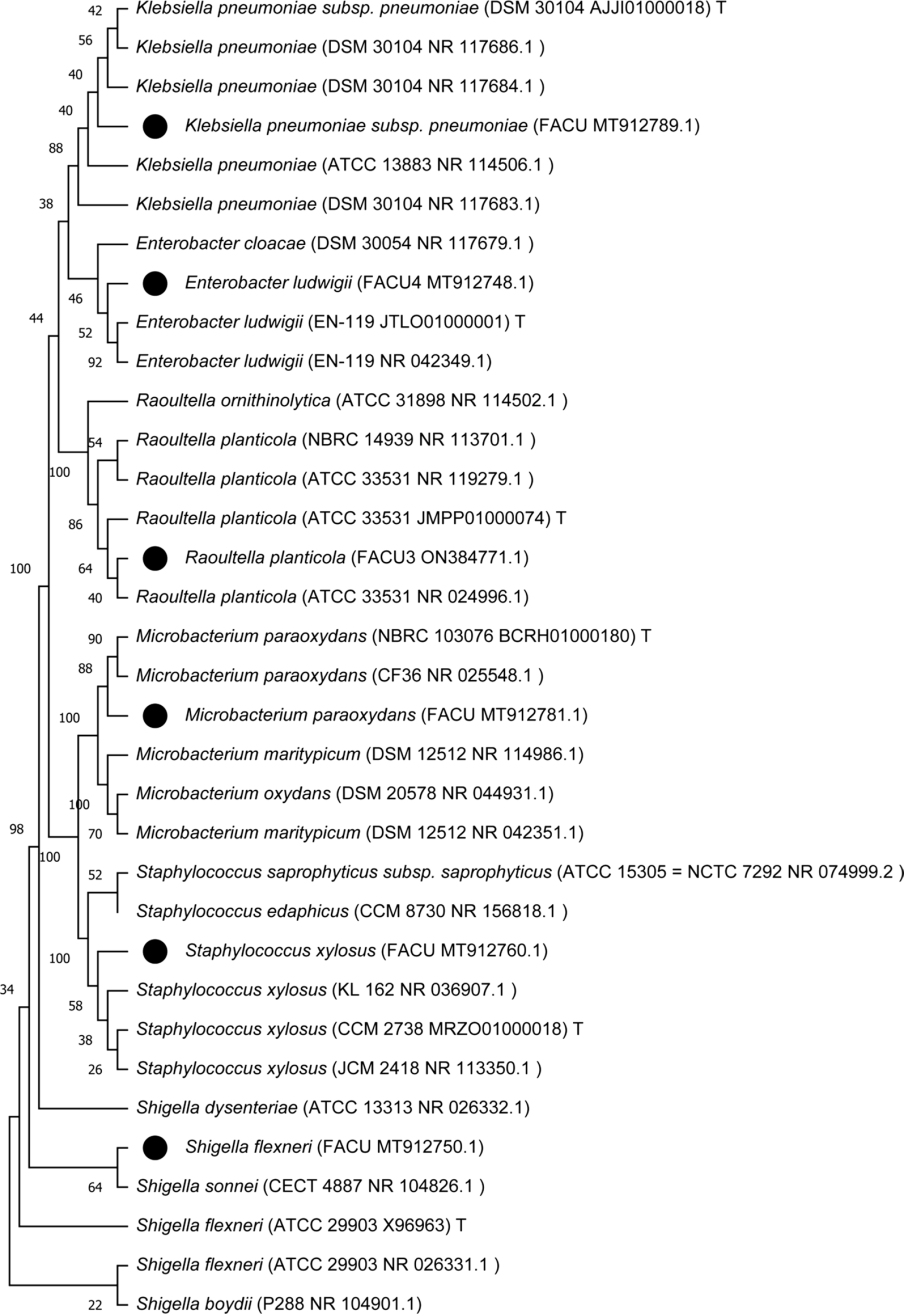
The phylogenetic tree of six Pb resistant strains utilizing16S rRNA gene sequence. Bootstrapping was performed for tree with 1000 replicates. Phylogenetic analyses were conducted in MEGA 11.

Morphological characterizations of the selected isolates were displayed in Additional file 1: Table S2. The six lead resistant isolates were molecularly identified using universal primers of *16S rRNA* gene. About 1500 bp of the *16S rRNA* gene was amplified and sequenced. The nucleotide sequence resulting from the six bacterial isolates *16S rRNA* gene sequencing was compared with the GenBank databases utilizing BLAST tools. The partial *16S rRNA* gene sequences from the six isolates were submitted to the GenBank database. All strains were deposited to NCBI with strain code FACU such as *Enterobacter ludwigii* FACU 4, *Shigella flexneri* FACU, *Microbacterium paraoxydans* FACU, *Klebsiella pneumoniae* subsp. pneumonia FACU, *Raoultella planticola* FACU 3 and *Staphylococcus xylosus* FACU under different accession numbers with the percentage of similarity (Table 1). The similarities of the six strains were ranged between 98.46 to 99.91%. The *16S rRNA* gene sequences of the strains and their closely related strains were used for the phylogenetic trees construction (Fig. 1). The bacterial strains were deposited and available in Culture Collection Ain Shams University (CCASU WDCM1186, Cairo-Egypt), under the numbers CCASU-2022-34 to CCASU-2022-39 for *E. ludwigii* FACU 4, *S. flexneri* FACU, *M. paraoxydans* FACU, *K. pneumoniae* subsp. pneumonia FACU, *R. planticola* FACU 3 and *S. xylosus* FACU, respectively.

**Table 1:** Top-hit taxon, similarity percentage and accession numbers of the selected bacterial isolates

Isolate code	Top-hit Taxon	Similarity (%)	Accession number
L3	*Enterobacter ludwigii*	99.27	MT912748
L4	*Shigella flexneri*	99.91	MT912750
L7	*Microbacterium paraoxydans*	99.56	MT912781
L8	*Klebsiella pneumoniae subsp. pneumoniae*	99.59	MT912789
L16	*Raoultella planticola*	99.7	ON384771
L17	*Staphylococcus xylosus*	98.46	MT912760

### Evaluating the ability of the selected bacterial strains to resist lead

**Fig. 2:**
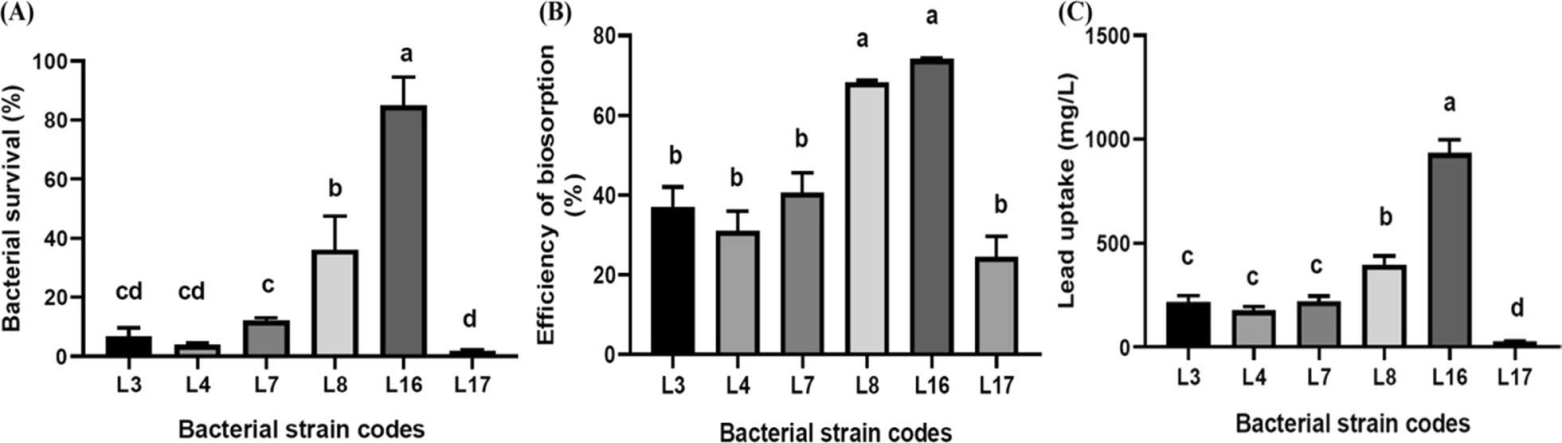
Evaluating the ability of the selected bacterial strains to resist lead through determination of bacterial survival (%) on lead stress (**A**), effeciency of biosorption (%) (**B**) and lead uptake (mg/L) (**C**). L3: *Enterobacter ludwigii*, L4: *Shigella flexneri*, L7: *Microbacterium paraoxydans*, L8: *Klebsiella pneumoniae subsp. pneumoniae*, L16: *Raoultella planticola* and L17: *Staphylococcus xylosus*

Bacterial survival (%), efficiency of biosorpion (%) and Pb uptake (mg/L) were utilized to evaluate the ability of Pb resistance for the six bacterial strains (Fig. 2). One way ANOVA and LSD tests showed significant differences between these strains. It was found that *R. planticola* (L16) strain was the highest significant percentage (84.8%) of bacterial survival on LB supplemented with 2000 ppm lead acetate with efficiency of biosorption approximately (73%) which can uptake 933.3 mg/L. *K. pneumoniae subsp. pneumoniae* (L8) had a moderate bacterial survival (36%) with efficiency of biosorption approximately (68%) which can uptake 330 mg/L on the same conditions. In contrast to, the remaining four strains *E. ludwigii* (L3), *S. flexneri* (L4), *M. paraoxydans* (L7) and *S. xylosus* (L17) showed a lowest significant percentage of bacterial survival (12, 1.7, 3.8 and 6.7%) with efficiency of biosorption approximately ( 37, 31, 40 and 24%) which can Pb uptake (215.3, 175.8, 219.15 and 25.7 mg/L) respectively.

### Investigation of morphological lead response of bacterial strains by TEM

**Fig. 3:**
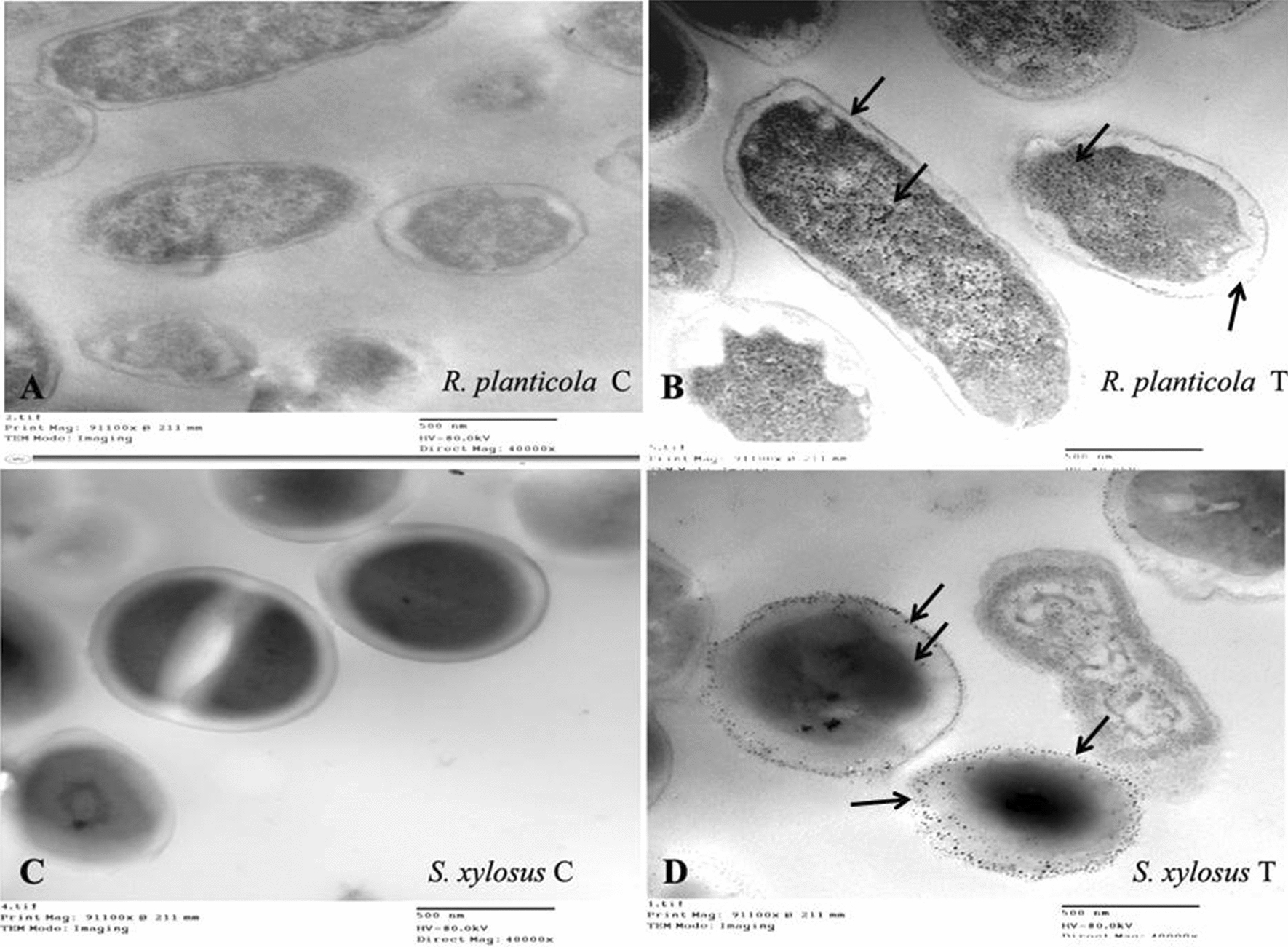
Transmission electron micrograph with magnification (40000×) for *R. planticola* FACU 3 and *S. xylosus* FACU in liquid LB medium supplemented with 2000 ppm Pb and without as control. **A** and **B** represent *R. planticola* while **C** and **D** represent* S. xylosus.* Arrows indicate to the metal accumulation regions

TEM was utilized to detect the morphological changes of the bacterial cell during exposure to Pb stress and study the difference between the highest and lowest bacterial strains *R. planticola* and *S. xylosus* in cell viability, efficiency of biosorption and lead uptake as shown in Fig. 3. In the case of *R. planticola*, there was slight difference in the cell size, shape and thickness of cell wall between control and lead treated cells. It was observed that there were black spots accumulated inside the cell along the cytoplasm in treated cells only as shown in Fig. 3B unlike control cells as in Fig. 3A. On the other hand, there was a clear difference in the cell shape and its status in *S. xylosus*. We found that in the treated cells, the cell wall may be disappeared and the cell morphology was deformed differently from the control cells had a defined cell morphology as in Fig. 3C. Also, there were black spots out the cells, shrinkage in cytoplasm and became darker at the middle of the cell as shown in Fig. 3D.

### Genome analysis

**Fig. 4:**
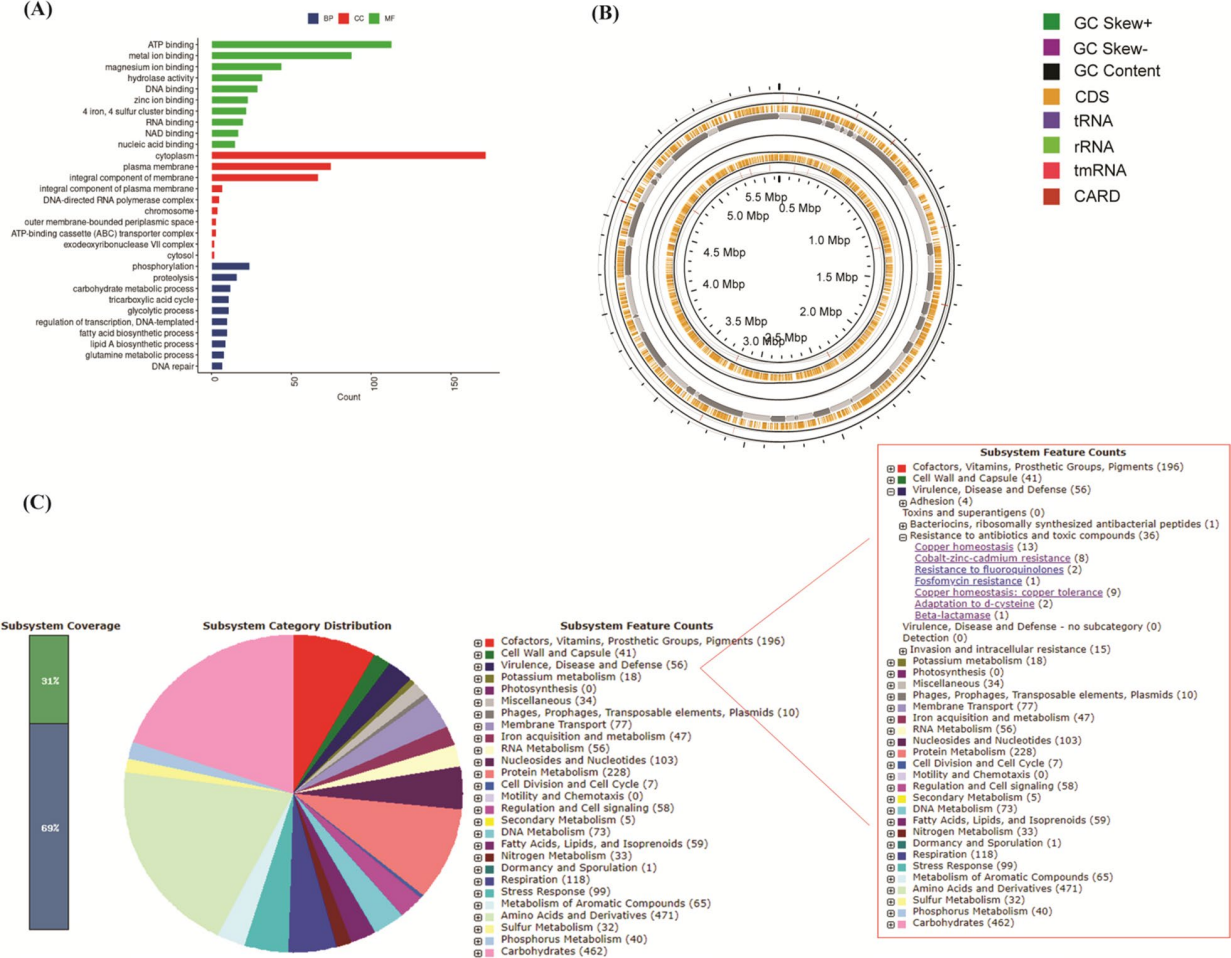
The gene ontology biological process, circular graphical and subsystem distribution of *R. planticola* FACU 3. **A** Top 10 Biological process (BP), Cellular component (CC) and molecular function gene ontologies generated by UniprotR, **B** the circular graphical display of the distribution of *R. planticola* FACU 3 genome which includes CDS on the forward strand, CDS on the reverse strand, RNA genes, GC content, positive and negative GC skew and antibiotic resistance genes. The figure was prepared by CGView circular genome visualization tool and **C** the subsystem coverage and category distribution of *R. planticola* FACU 3 genome utilizing RAST. The pie chart demonstrates the counts for each subsystem feature and the subsystem coverage. Genes for each Subsystem Category were displayed in brackets

**Fig. 5:**
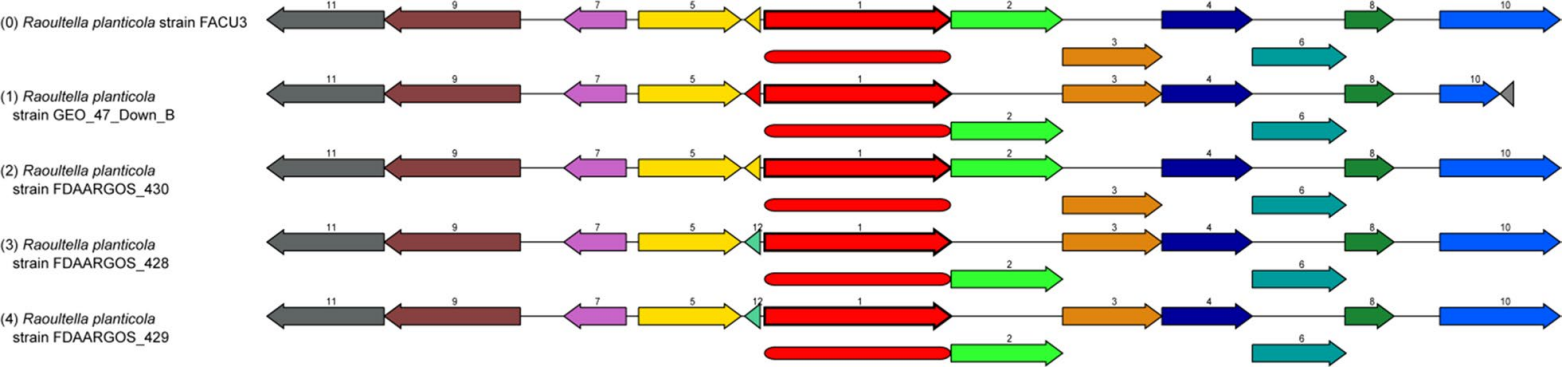
Nickel resistance gene cluster: The chromosomal sequence of the focus gene (top) was compared with three similar organisms. The graphic depicts the focus gene, which is red and numbered 1. Sets of genes with similar sequence are grouped with the same number and color (1- Nickel ABC transporter, substrate-binding protein NikA, 2- Nickel ABC transporter, permease protein NikB, 3- Nickel ABC transporter, permease protein NikC, 4- Nickel ABC transporter, ATP-binding protein NikD, 5- Transcriptional regulator, LysR family, 6- Nickel ABC transporter, ATP-binding protein NikE,7- Phenolic acid decarboxylase, 8- Nickel responsive regulator NikR, 9-UDP-4-amino-4-deoxy-L-arabinose–oxoglutarate aminotransferase, 10- Mg/Co/Ni transporter MgtE, CBS domain-containing, 11- Undecaprenyl-phosphate 4-deoxy-4-formamido-L-arabinose transferase and 12-hypothetical protein)

The results indicate that *R. planticola* FACU 3 had the highest ability for Pb resistance, so it was selected for WGS studies. WGS of *R. planticola* FACU 3 was carried out utilizing the illumine sequencing platform, and the results displayed that the assembled genome yielded 50 contigs, with a size of 5,648,460 bp and GC content 55.8% (Table 2). The contigs have an N_50_ value of 158,943 bp and an L_50_ value of 11. Three different tools Prokka, Bakta and RAST were utilized for genome annotation. The annotation results were combined to cover throughout the genome and identified 5526 CDS, 75 tRNA and 4 rRNA. Among the protein coding genes, 818 hypothetical proteins and 4707 proteins were assigned with functional assignments (Fig. 4A). The functional assignments proteins included 1453 proteins with Enzyme Commission (EC) numbers, 1212 with Gene Ontology (GO) assignments and 1060 proteins that were mapped to KEGG pathways (Fig. 4B). About 561 annotated genes showed homology to known transporters genes according to the transporter classification database (TCDB), 53 genes displayed homology to antibiotic resistant genes based on CARD and 305 genes were drug target genes according to DrugBank (Table 2). The statistics of the genome, the detailed properties and the genes distribution into COG functional categories are summarized in Table 3 and Fig. 4C. Function annotation of identified protein-encoding genes in *R. planticola* FACU 3 was performed using COG, a number of heavy metal resistant genes and gene clusters were found (Table 3). The genome also harbors system genes and clusters involved in the resistance and transport of zinc, cadmium, lead, and nickel, cobalt, copper, mercury and silver. In addition to identifying two plasmids having FII and Col replicons which had 97.36 and 96.12 identity with coverage 100 and 98.47 respectively in this genome. PATRIC genome annotation service was utilized for antimicrobial resistant (AMR) genes screening. The results displayed that *R. planticola* FACU3 genome contained several AMR genes (Table 4). The majority of *R. planticola* FACU3 AMR genes was involved in conferring resistance via efflux pumps and modified antibiotic targets. Analysis of *R. planticola* FACU3 resistome using the RGI tool identified 1 perfect hits and 24 strict hits as provided in Additional file 1: Table S3. The presence of multiple resistant genes is expected to make the strain resistant to carbapenem and cephalosporin. From gene annotation nickel resistance gene cluster was observed as a model for AMR genes in *R. planticola* FACU3 (Fig. 5). The chromosomal sequence of the focus gene was compared with three similar organisms *R. planticola* strain *GEO, R. planticola* strain *FDAARGOS* 430 and *R. planticola* strain *FDAARGOS* 428. Moreover, this genome harbors many PGPGs such as genes that participate in IAA production, phosphate solubilization, acetoin and butanediol synthesis, chitinase production, phenazine production, trehalose metabolism, 4-hydroxybenzoate production, heat shock proteins, cold shock proteins, H_2_S production, peroxidases, catalases, siderophore production, superoxide dismutase and denitrification (Table 5).

**Table 2:** Genomic features of *R. planticola* FACU 3 genome

Features	Term
Contigs	52
GC content	55.8%
Contig L50	11
Genome Length	5,648,460 (bp)
Contig N50	158,943
Number of Subsystems	386
CDS	5526
tRNA	75
rRNA	4
Transporter	561
Antibiotics	53
Drug target	305
Hypothetical proteins	818
Proteins with functional assignments	4707
Proteins with EC number assignments	1453
Proteins with GO assignments	1212
Proteins with Pathway assignments	1060
Bio sample	SAMN29720013
Bio project	PRJNA858473
Accession number	JANEWN000000000

**Table 3:** Genes/gene clusters of heavy metal resistance in *R. planticola* FACU 3

Genes	Function	length	Heavy metal resistance	Locus tag
*zntA*^b^	Zinc/cadmium/lead-transporting P-type ATPase	2199	Zn, Cd, Pb	NM584_04589
*chrR*^c^	Quinone reductase	567	Cr	NM584_03451
*arsA*	Arsenical pump-driving ATPase	1752	As	NM584_05684
*arsB*^c^	Arsenical pump membrane protein^a^	1293 1290 1167	As	NM584_01807 NM584_05685 NM584_05812
*arsC*^c^	Arsenate reductase^a^	426 426 429	As	NM584_01808 NM584_05686 NM584_05813
*arsH*^c^	NADPH-dependent FMN reductase^a^	699 723	As	NM584_01805 NM584_05814
*arsD*^c^	Arsenical resistance operon trans-acting repressor	363	As	NM584_05683
*arsR*^c^	Arsenic resistance transcriptional regulator	354	As	NM584_05653
*rcnR*^b^	Transcriptional repressor RcnR	272	Ni, Co	NM584_00068
*rcnB*^b^	Nickel/cobalt homeostasis protein RcnB	318	Ni, Co	NM584_02529
*hoxN*^b^	High-affinity nickel transport protein	1014	Ni	NM584_01839
*cnrA*^b^	Nickel and cobalt resistance protein	3183	Ni, Co	NM584_00460
*nikA*^c^	Nickel-binding periplasmic protein^a^	1569 1623	Ni	NM584_03175 NM584_04730
*nikB*^c^	Nickel transport system permease protein	945	Ni	NM584_03176
*nikC*^c^	Nickel transport system permease protein	834	Ni	NM584_03177
*nikD*^c^	Nickel import ATP-binding protein	765	Ni	NM584_03178
*nikE*^c^	Nickel import ATP-binding protein	792	Ni	NM584_03179
*nikR*^c^	Nickel-responsive regulator	417	Ni	NM584_03180
*zitB*^b^	Zinc transporter ZitB	939	Zn	NM584_00719
*zntB*^b^	Zinc transport protein ZntB^a^	1029 934	Zn	NM584_02816 NM584_04340
*zntR*^b^	HTH-type transcriptional regulator	426	Zn	NM584_05551
*znuA*^b^	High-affinity zinc uptake system protein ZnuA	945	Zn	NM584_01443
*znuC*^b^	Zinc import ATP-binding protein ZnuC	753	Zn	NM584_01442
*znuB*^b^	High-affinity zinc uptake system membrane protein ZnuB	786	Zn	NM584_01441
*yeiR*^b^	Zinc-binding GTPase YeiR	978	Zn	NM584_02585
*zur*^b^	Zinc uptake regulation protein	516	Zn	NM584_05063
*ftsH*^b^	ATP-dependent zinc metalloprotease FtsH	1944	Zn	NM584_04403
*zupT*^b^	Zinc transporter ZupT	771	Zn	NM584_02221
*copA*^b^	Copper-exporting P-type ATPase Copper resistance protein A	2502 1818	Cu Cu	NM584_01046 NM584_05521
*copB*^b^	Copper resistance protein B	897	Cu	NM584_05520
*pcoC*	Copper resistance protein C	381	Cu	NM584_05519
*copD*^b^	Copper resistance protein D	930	Cu	NM584_05518
*copR*^b^	Transcriptional activator protein^a^	681 702	Cu	NM584_05517 NM584_01018
*sasA*^b^	Adaptive-response sensory-kinase	1401	Cu	NM584_05516
*pcoE*^b^	putative copper-binding protein PcoE	435	Cu	NM584_05515
*cutC*^b^	Copper homeostasis protein CutC	744	Cu	NM584_01429
*cueO*^b^	Blue copper oxidase	1626	Cu	NM584_03744
*cueR*^b^	HTH-type transcriptional regulator CueR	411	Cu	NM584_01041
*cusA*^b^	Cation efflux system protein CusA^a^	3156 3146	Cu, Ag	NM584_03841 NM584_05527
*cusB*^b^	Cation efflux system protein CusB^a^	1278 1293	Cu, Ag	NM584_03840 NM584_05528
*cusF*^b^	Cation efflux system protein CusF^a^	348 354	Cu, Ag	NM584_03839 NM584_05529
*cusC*^b^	Cation efflux system protein CusC^a^	1482 1494 1386	Cu, Ag	NM584_02048 NM584_03838 NM584_05530
*cusR*^b^	Cation efflux system protein CusR^a^	684 681	Cu, Ag	NM584_03837 NM584_05531
*cusS*^b^	Sensor histidine kinase CusS^a^	1452 1467	Cu, Ag	NM584_03836 NM584_05532
*silE*^b^	Silver-binding protein^a^	450 432	Ag	NM584_05522 NM584_05533
*silP*^b^	Silver exporting P-type ATPase	2442	Ag	NM584_05525
*merR*^b^	Transcriptional regulator^a^	399 456	Hg	NM584_00139 NM584_02915

Numbers depict relevant contigs, Locus tags are from Prokka annotation

^a^Some genes exist in multiple copies and locations, ^b^Genes located on chromosome, ^c^Genes located on plasmid

**Table 4:** The AMR genes annotated in this genome and corresponding AMR mechanism

Genes	ABR mechanism
*katG*	Antibiotic activation enzyme
*PLA* family	Antibiotic inactivation enzyme
*marA, marB, marR*	Antibiotic resistance gene cluster or operon
*alr, ddl, dxr, EF-G, EF-Tu, folA, Dfr, folP, gyrA, gyrB, inhA, fabI, Iso-tRNA, kasA, MurA, rho, rpoB, rpoC, S10p, S12p*	Antibiotic target in susceptible species
*bcrC*	Antibiotic target protection protein
*acrAB-TolC, acrAD-TolC, acrZ, emrAB-TolC, emrD, macA, macB, mdfA/Cmr, mdtABC-TolC, mdtL, sugE, tolC/OpmH*	Efflux pump conferring antibiotic resistance
*gdpD, pgsA*	Protein altering cell wall charge conferring antibiotic resistance
*oprB*	Protein modulating permeability to antibiotic
*acrAB-TolC, emrAB-TolC, H-NS, oxyR*	Regulator modulating expression of antibiotic resistance genes

**Table 5:** List of plant growth promoting genes in *R. planticola* FACU3

Genes	Plant growth promotion properties
*ipdC*	IAA production
*pstS, A, B, C*	Phosphate solubilization
*budA, B, C* and *poxB*	Acetoin and butanediol synthesis
*chiA*	Chitinase production
*phzS*	Phenazine production
*treC, B, otsA, B* and *sugA*	Trehalose metabolism
*ubiD, dhdA, entA, pcak* and *pobA*	4-hydroxybenzoate production
*hspQ, hslJ, R* and *ibpA, B*	Heat shock proteins
*cysZ, K, M, A, W, T, P, L, G, J, I, H, D, C, S* and* N*	H_2_S production
*cpsA, B, C, E, D, LA* and ydfK	Cold shock proteins
*cpo, efeB, butE, katG* and *yfeX*	Peroxidases
*kat E,G* and *ydbD*	Catalases
*yfiZ, yusV* and *fiu*	Siderophore production
*sodA, B*	Superoxide dismutase
*nirD, fnr, fdnGHIT, narGHIJKLUVWXYZ*	Denitrification

## Discussion

In this study, 30 bacterial isolates for lead resistance bacteria were isolated and purified from three different locations. EC is a measure of the conducting capacity of water and it is measured by ionic found in the water. The presence of a slightly high value of electrical conductivity in the water sample displays that contaminations due to dissolve ions are high, because electrical conductivity is directly proportional to the total dissolved solids. This may be due to the geology of the study area and soil type or the wastes entering from the surrounding water sources (Christine et al. 2018). The pH of the collected samples was observed slightly acidic while the EC was noticed as low conductivity which agreement with Zouch et al. (2018). The findings specify that the collected industrial wastewater and sediment samples were enormously polluted with high concentrations of different heavy metals. Six bacterial isolates were selected based on the highest ability for Pb resistance due to their cell survival, Pb removal efficiency and Pb uptake. From our results, we can assume that the low significant percentage of bacterial survival on lead stress, efficiency of biosorption and lead uptake may be due to these strains reach to toxicity level so Pb stress had an impact on cell survival. Toxicity can increase uptake due to the toxicity increases cell membrane permeability if the toxic pressure is not sufficient to kill the organisms (Odokuma and Akponah 2010). So, *R. planticola* FACU 3 (L16) can be considered as the best one because it didn’t have a negative impact from lead stress on cell viability with higher efficiency.

The morphological and biochemical tests were used for bacterial identification. The six isolates were identified using *16S rRNA* genes as the study mentioned before. These strains were well studied as heavy metals resistant bacteria for example, *Enterobacter ludwigii* is well known as plant growth promoting bacteria of wheat and rice under mercury, zinc, cadmium and nickel stress and produce biofilm to absorb copper, nickel and lead (Singh et al. 2018; Adhikari et al. 2020; Haque et al. 2021). *Staphylococcus* species was reported that have ability to tolerate chromium and lead (Ugur and Ceylan 2003). Manzoor et al. (2019) reported that *Microbacterium sp.* and *Klebsiella sp.* are lead resistant plant growth promoting bacteria with high efficiency. *Shigella* sp. was investigated its ability to resist mercury, silver and arsenic (Sultan et al. 2020). In addition, *R. planticola* can serve as multidrug and heavy metals resistant bacteria (Koc et al. 2013). It is well studied the ability of *R. planticola* to resist heavy metals. Koc et al. (2013) tested the ability of *R. planticola* isolated from surface water in Turkey to resist lead up to 1200 ppm and other heavy metals. Also, it was reported that *R. planticola* (R3) can grow at 1500 ppm lead and was reported that it can remove several heavy metals like lead, manganese, cadmium, copper, zinc and nickel (Bowman et al. 2018). *Raoultella planticola* (VIP) was determined the minimum inhibition concentration reached to 350 ppm and completely removed lead at 90 h (Eltarahony et al. 2021) while the MIC for *R. planticola* FACU 3 was to 2800 ppm. In this study, TEM was used to investigate the morphological Pb response of two bacterial strains, the highest strain (*R. planticola* FACU 3) and lowest strains (*S. xylosus* FACU) in cell viability, efficiency of biosorption and Pb uptake. The result showed that there was a little difference in the cell size, shape and thickness of cell wall in treated strain compared with the control. Also it was observed that there were black spots accumulated inside the cell along the cytoplasm in treated cells only. Our results was in agreed with the results from Oves et al. (2016) and Nies (1999) who supposed that the heavy metals resistance may be due to the bacterial cell viability not the effect of cell enzymatic activity. They reported that the cell viability when exposed to chromium due to its toxic effect on bacterial cell morphology and not because of enzymatic inhibition or the membrane damage. After bacterial chromium uptake into the cell, chromium was associated with extracellular interactions and caused cell morphology deformation.

To further investigate the Pb accumulation mechanism and metabolic pathway, we carried out WGS of *R. planticola* FACU 3. The genome size was similar to the draft genome reported for the strain HH15, R1Gly and CHB but with GC content less than our strain FACU (Jothikumar et al. 2014; Schicklberger et al. 2015; Kang et al. 2021). FACU 3 contains high *tRNA* genes content which may reflect the adaptation of cell to extreme conditions (Wu et al. 2011) through controlling gene expression in microorganisms under highly variable environment (Rodríguez-Rojas et al. 2016). The annotated CDS were done using COG database, with the main focus on general function prediction, amino acid transport and metabolism, transcription, heavy metals resistance genes. A number of heavy metal resistant genes and gene clusters were found. The results also annotated genes and clusters involved in the resistance and transport of zinc, cadmium, lead, and nickel, cobalt, copper, mercury and silver. This is agreed with previous published draft genome of *R. planticola* (Jothikumar et al. 2014)*. Raoultella planticola* FACU 3 contains z*ntA* gene which work as zinc/cadmium/lead-transporting P-type ATPase (Naik and Dubey 2011; Guo et al. 2022). *zntA* is significantly induced by lead and cadmium not by zinc only, its expression is uprgulated and is mediated by *zntR* (Binet and Poole 2000). z*ntR* and *zntA* genes are not adjacent to each other but are located on different locations of the chromosome (Baya et al. 2021). So, this supports our results of *R. planticola* FACU 3’s ability to resist lead. Additionally, FACU 3 has znt B which work as a zinc efflux pathway (Worlock and Smith 2002). zit B plays an important role in zinc homeostasis at low concentrations of zinc however znt A at high concentrations (Grass et al. 2001). *znuABC* operon and *zur* gene was found in *R. planticola* FACU 3 and it was reported that zinc transporter encoded by the *znuABC* gene cluster and is regulated by *zur* gene product in response to the intracellular zinc concentration (Patzer and Hantke 2000). ZupT mediates zinc uptake and may also transport other divalent cations such as copper and cadmium ions (Grass et al. 2002). YieR participate in metal hemostasis (Blaby-Haas et al. 2012). From previous data, it was revealed that *R. planticola* FACU 3 has various gene system and gene clusters for zinc resistance.

*R. planticola* FACU 3 has *nikABCDE–nikR* operon which can control nickel concentration in the cell (Binet and Poole 2000) as provided in Fig. 5 the nickel resistance gene cluster in comparison with three similar organism via PATRIC. *Rcn*-operon (*rcn R, B*) encoded for nickel–cobalt efflux system (Blaha et al. 2011). As well as, *hox N* gene existed in *R. planticola* FACU 3 and it is known as high-affinity nickel transport protein and mediate in nickel transport (Wolfram et al. 1995). So, *R. planticola* FACU 3 has different types of high affinity nickel transport system. *R. planticola* FACU 3 harbors *ars RDABC* operon and *arsH* which was encoded for arsenic resistance (Carlin et al. 1995; Vorontsov et al. 2007; Yang et al. 2012). It is worth noting that the presence of *quinone reductase* gene (*chrR*) helps in reducing of chromate thus in chromate bioremediation (Eswaramoorthy et al. 2012; Paul et al. 2020). There are gene systems/gene cluster encoded for copper hemostasis like *cop* operon, *cutC, cue O* and *cue R* in addition to *cus* operon and *pco/sil* operon which participates in silver efflux system (Franke et al. 2003; Gudipaty et al. 2012) and found on the chromosome as several studies reported that these genes are regularly be located on the chromosome of *Enterobacteriaceae* species not restricted to plasmid (Baya et al. 2021). The presence of *mer R* which is a transcriptional regulator for mercury resistance may be evidence of *R. planticola* FACU 3 may be mercury resistant bacteria (Brown et al. 2003). These heavy metal genes participate in bacterial survival under heavy metals stress as observed in *Rhodobacter sphaeroides* which used as a model bacterium to explore the heavy metal bioremediation (Johnson et al. 2019). They analysed the distribution of heavy metal genes across bacterial species and found that there were about 170,000 heavy metal related genes with a majority of the genes found in Proteobacteria (46%) and Terrabacteria (39%). As well as, *R. sphaeroides* genome contains a total of 375 heavy metal resistance genes. Also other previous studies reported that one bacteria strain could have between 28 to 55 heavy metal resistance genes (Abbaszade et al. 2020, Klonowska et al. 2020, Yang et al. 2022, Carro et al. 2022). The high percentage of genes related to heavy metal resistance in these bacteria suggests that the heavy metal resistance genes have possibly evolved multiple times; however the wide distribution of the heavy metal genes also supports the notion that many other bacterial species have acquired these genes by horizontal gene transfers (HGT) (Johnson et al. 2019).

Also, it was predicted the presence of genes encoded for resistance to antibiotics like fluoroquinolones, fosfomycin, β-lactamase the mdtABCDKLNO multidrug resistance cluster, and multidrug resistance efflux pumps. Potential antimicrobial compounds could be produced by microbes during bioremediation process (Abdelhadi et al. 2016; El-Arabi et al. 2018). From the previous results, it is revealed that *R. planticola* FACU 3 multi heavy metals resistance bacteria other than lead. Furthermore, the presence of AMR-related genes (even full length) in a given genome does not directly imply antibiotic resistant phenotype. It is important to consider specific AMR mechanisms and especially the absence/presence of single nucleotide polymorphism (SNP) mutations conveying resistance. So, from resistome analysis we can expect that this strain is resistant to carbapenem and cephalosporin and as known both antibiotic are classes of beta-lactam antibiotics. This confirmed by many studies which reported that antibiotic resistance genes can be influenced by ecosystem heavy metals contamination (Knapp et al. 2017). As well as the bacterial heavy metal resistance genes and antibiotic resistance genes can respond to the heavy metals inducement (Chen et al. 2019). It is worth to note that the presence of plant growth promoting genes in *R. planticola* FACU3 can participate in improving nutrient availability, oxidative stress resistance, suppression of biotic and abiotic stress. FACU3 genome contains *ipdC* gene which codes for indole pyruvate decarboxylase, an enzyme that produces indole acetic acid (IAA) from tryptophan (Straub et al. 2013). In this genome we also found the *trp* cluster (*trpA, B, C, R, and S*) genes involved in tryptophan biosynthesis. These genes may play a role in synthesis of tryptophan utilized in IAA hormone biosynthesis which helps in plant growth (Duca et al. 2014). The genome of FACU3 possessed genes encoding phosphate-specific transport system (*pst*) operon which participate in solubilization of mineral phosphates in soil (Brito et al. 2020). In addition, genes involved in hydrogen sulfide (H_2_S) biosynthesis are present in FACU3 which takes part in seed germination and increasing of plant growth (Dooley et al. 2013). As well as genes *budA, B, C* and *poxB* were identified in FACU3 which are involved in the production of acetoin and 2,3-butanediol which influence the plant growth promotion (Ryu et al. 2003). Furthermore, FACU3 genome coded for several genes that encode catalases, peroxidases and superoxide dismutase all of which alleviate oxidative stress in plants (Rai et al. 2013). Also, the *phzS* and (*ubiD, dhdA, entA, pcaK* and *pobA)* genes encoded for phenazine synthesis, 4-hydroxybenzoate synthesis and respectively are existed in FACU3 genome and participate in plant growth promoting and reduction of osmotic stress (Yuan et al. 2020) and plant defense and communication (Bhattacharya et al. 2010) respectively. Besides these, genes for heat shock tolerance, cold shock tolerance and trehalose production that enable bacteria to survive abiotic stress were identified. Moreover, *chiA* gene was identified which encoded for chitinase production responsible for the nutrient cycling of chitin so this bacteria can be used in biocontrol (Veliz et al. 2017). Production and secretion of siderophores is an important metabolic feature utilized by bacteria because iron bioavailability is poor so acquisition of iron is essential for bacterial survival (Miethke and Marahiel 2007). FACU3 genome contains *yfiZ, yusV* and *fiu* genes which participate in iron acquisition (Grinter and Lithgow 2019; Endicott et al. 2020). From our findings we can consider *R. planticola* FACU3 as a plant growth promoting bacteria besides its ability to resist multi heavy metals. The draft genome of *R. planticola* strain FACU 3 provides an insight into the genomic basis of its heavy metal resistance ability, multidrug resistance, antibiotic resistance and plant growth promoting traits. Also it provides great information about its ability to resist more kinds of heavy metal. In conclusion, this strain could be utilized in different heavy metals bioremediation or the good source of heavy metals resistant genes besides plant growth promoting genes which make it plant growth promoting bacteria. The *R. planticola* FACU 3 draft genome can be utilized as a base/reference sequence to explore and map specific genes related to Pb and other heavy metals genes. It could be a valuable resource to conduct comparative analyses among different species related to *R. planticola* FACU 3, which may have similar heavy metals resistance properties.

## Supplementary Information

**Additional file 1: Table S1.** The physicochemical analysis and heavy metal concentrations of the collected samples. **Table S2.** Morphological, microscopic and biochemical characteristics of the six bacterial isolates. **Table S3.** Analysis of *R. planticola* FACU3 resistome using the RGI tool. **Figure S1.** The MIC and MTC. A: for the different three collection locations and B: for the thirty selected lead resistant isolates.

## Data Availability

This *R. planticola* FACU3 draft genome sequences was deposited at NCBI GenBank under the Accession JANEWN000000000.

## Abbreviations

*R. planticola* FACU 3: *Raoultella planticola* FACU 3
Pb: Lead
MIC: Minimum inhibitory concentration
MTC: Maximum tolerance concentration
TEM: Transmission electron microscopy
PGPGs: Plant growth-promoting genes
IAA: Indole acetic acid
EPS: Extra cellular polymeric substances
As: Arsenic
Cd: Cadmium
Hg: Mercury
CBA: Capsule biogenesis assembly
CDF: Cation diffusion facilitator
EPSs: Exopolysaccharides
WGS: Whole-genome sequencing
KEGG: Kyoto encyclopedia of genes and genomes
GO: Gene ontology
NR: Non-redundant
COG: Clusters of orthologous genes
Cr: Chromium
Cu: Copper
Fe: Iron
Mg: Manganese
Ni: Nickel
Zn: Zinc
CFU: Colony forming unit
LB: Luria Bertani
UV: Ultraviolet
RGI: Resistance gene identifier
CARD: Comprehensive antibiotic resistance database
ANOVA: One way analysis of variance
LSD: Least significant difference
EC: Electric conductivity
US EPA: United States environmental protection agency
EC: Enzyme commission
TCDB: Transporter classification database
AMR: Antimicrobial resistant
SNP: Single nucleotide polymorphism
PST: Phosphate-specific transport system
NBNE: National biotechnology network of expertise
ASRT: Academy of science research and technology
CC: Cellular component
BP: Biological process
MF: Molecular function
