# Supplementary material for: Draft genome of Raoultella planticola, a high lead resistance bacterium from industrial wastewater
Source: AMB Express. 2023 Jan 30;13:14. doi: 10.1186/s13568-023-01519-w (PMC9885416; doi:10.1186/s13568-023-01519-w)
Supplement: Supplementary file 1 — Additional file 1: Table S1. The physicochemical analysis and heavy metal concentrations of the collected samples. Table S2. Morphological, microscopic and biochemical characteristics of the six bacterial isolates. Table S3. Analysis of R. planticola FACU3 resistome using the RGI tool. Figure S1. The MIC and MTC. A: for the different three collection locations and B: for the thirty selected lead resistant isolates. [file 13568_2023_1519_MOESM1_ESM.doc]

Table S1 The physicochemical analysis and heavy metal concentrations of the collected samples

| **Locations** | **pH** | **EC**  **(mS/cm)** | **Heavy metal contents in collected samples (mg/L) for wastewater sample or (mg/Kg) for sediment sample*** | | | | | | | | |
| --- | --- | --- | --- | --- | --- | --- | --- | --- | --- | --- | --- |
|  |  |  | **As** | **Cd** | **Cr** | **Cu** | **Fe** | **Mn** | **Ni** | **Pb** | **Zn** |
| **4th industrial zone, Borg Elarab** | 6.6 ± 0.1 | 1.6±  0.1 | 3.1 ± 0.1 | 0.27  ± 0.1 | 0.17 ±  0.1 | 0.3 ± 0.1 | 3.2±  0.1 | 0.08± 0.01 | 0.13± 0.06 | 0.7±  0.1 | 3.2± 0.1 |
| **El Rahawy drain (sediment)** | 6.4 ± 0.1 | 1.1±  0.1 | 1.5 ± 0.1 | 0.05± 0.01 | 27.5 ±  0.1 | 32.5 ± 0.1 | 7500 ± 100 | 450±  10 | 21.75± 0.1 | 6.25±  0.1 | 7.5± 0.1 |
| **El Rahawy drain (wastewater)** | 6.8 ± 0.1 | 0.95 ± 0.1 | 0.001 ± 0 | 0.001 ± 0 | 0.06 ± 0.01 | 0.6 ± 0.1 | 11.5± 0.1 | 0.27± 0.1 | 0.001± 0 | 0.001± 0 | 0.15± 0.1 |
| **The US EPA standard** |  |  | 0.01 | 0.005 | 0.05 | 1.3 | 0.3 | 0.05 | 0.02 | 0.01 | 5 |
| *These physicochemical analysis and heavy metal concentrations of industrial wastewater samples were analyzed in triplicates and the results were described as mean with standard deviations (±SD). | | | | | | | | | | | |

**Table S2 Morphological, microscopic and biochemical characteristics of the six bacterial isolates**

| **Items** | **L3** | **L4** | **L7** | **L8** | **L16** | **L17** |
| --- | --- | --- | --- | --- | --- | --- |
| Gram | **-** | **-** | **+** | **-** | **-** | **+** |
| shape | **Rod** | **Rod** | **Rod** | **Rod** | **Rod** | **Coccus** |
| Motility | **+** | **-** | **+** | **-** | **-** | **+** |
| oxidase | **-** | **-** | **-** | **-** | **-** | **-** |
| Catalase | **+** | **+** | **+** | **+** | **+** | **+** |
| Urease activity | **–** | **-** | **-** | **+** | **+** | **+** |
| Indole production | **–** | **+** | **-** | **-** | **-** | **-** |
| Methyl red test | **–** | **+** | **+** | **-** | **-** | **+** |
| Voges-Prokauer reaction | **+** | **-** | **-** | **+** | **+** | **+** |
| Sucrose | **+** | **-** | **+** | **+** | **+** | **+** |
| Glucose | **+** | **+** | **+** | **+** | **+** | **+** |
| L-Arabinose | **+** | **+** | **+** | **+** | **-** | **+** |
| Lactose | **+** | **-** | **+** | **+** | **-** | **+** |
| D-Xylose | **+** | **-** | **-** | **+** | **+** | **+** |
| Raffinose | **+** | **+** | **-** | **+** | **+** | **-** |
| L-Rhamnose | **+** | **-** | **-** | **+** | **-** | **-** |
| D-Manntiol | **+** | **+** | **-** | **+** | **+** | **+** |
| D-Sorbitol | **+** | **+** | **-** | **+** | **-** | **-** |
| **Identification** | ***Enterobacter*** | ***Shigella*** | ***Microbacterium*** | ***Klebsiella*** | ***Raoultella*** | ***Staphylococcus*** |

| **Table S3 Analysis of *R. planticola* FACU3 resistome using the RGI tool** | | | | | | | | | |
| --- | --- | --- | --- | --- | --- | --- | --- | --- | --- |
| **RGI Criteria** | | **ARO Term** | **SNP** | **Detection Criteria** | **AMR Gene Family** | **Drug class** | **Resistance mechanism** | **% Identity of Matching Region** | **% Length of Reference Sequence** |
| Perfect | PLA-2a | |  | protein homolog model | PLA beta-lactamase | carbapenem, cephalosporin | antibiotic inactivation | 100 | 100 |
|  |  | |  |  |  |  |  |  |  |
| Strict | *Escherichia coli* EF-Tu mutants conferring resistance to Pulvomycin | | R234F | protein variant model | elfamycin resistant EF-Tu | elfamycin antibiotic | antibiotic target alteration | 97.71 | 96.33 |
|  |  | |  |  |  |  |  |  |  |
| Strict | *Escherichia coli* UhpT with mutation conferring resistance to fosfomycin | | E350Q | protein variant model | antibiotic-resistant UhpT | phosphonic acid antibiotic | antibiotic target alteration | 96.27 | 100 |
| Strict | OmpA | |  | protein homolog model | General Bacterial Porin with reduced permeability to peptide antibiotics | peptide antibiotic | reduced permeability to antibiotic | 96.07 | 95.19 |
|  |  | |  |  |  |  |  |  |  |


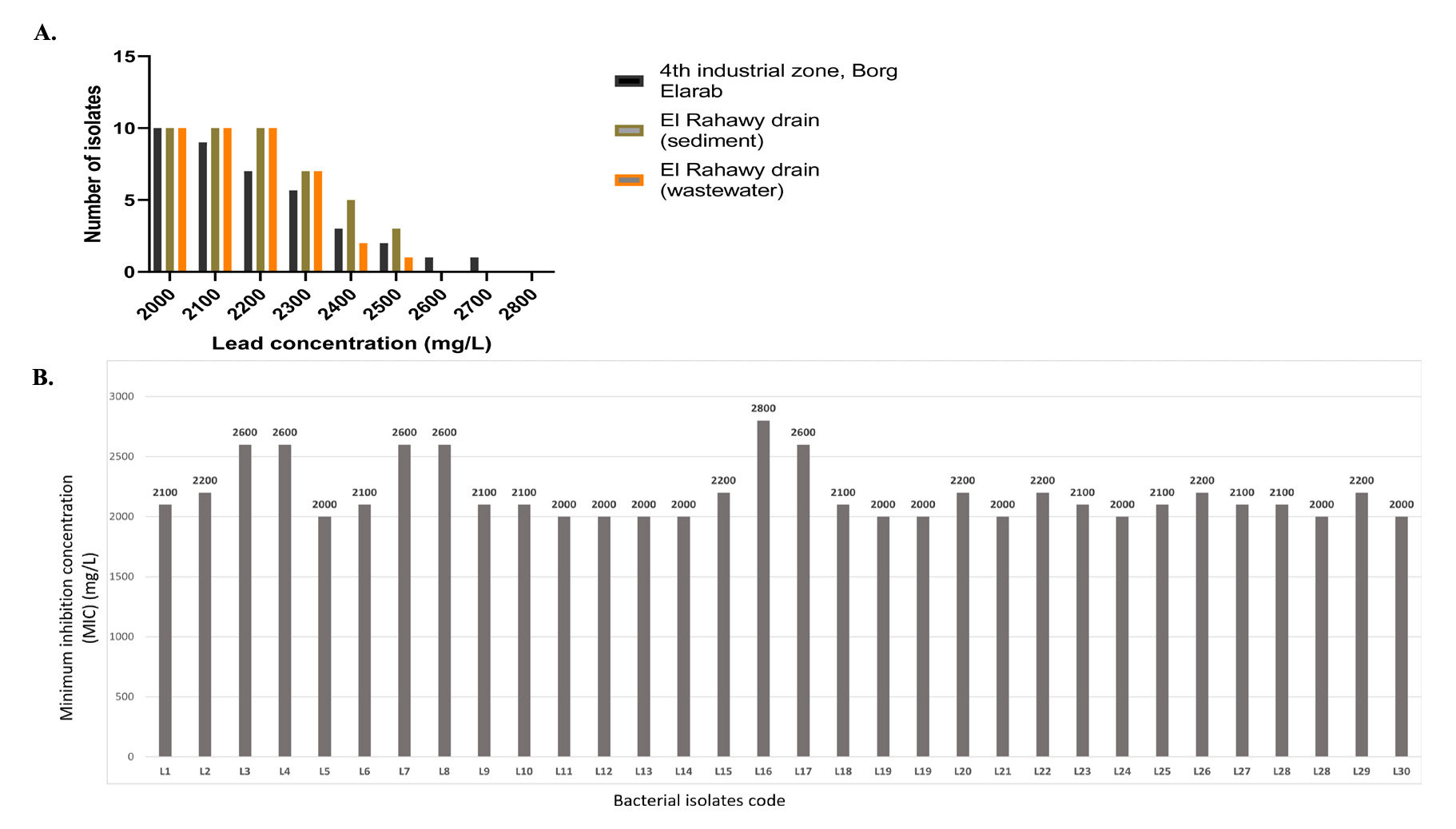


Fig. S1. The MIC and MTC. A: for the different three collection locations and B: for the thirty selected lead resistant isolates
